# The complete mitochondrial genome of *Menathais tuberosa* (Gastropoda, Neogastropoda, Muricidae) collected from Chuuk Lagoon

**DOI:** 10.1080/23802359.2016.1186516

**Published:** 2016-07-08

**Authors:** Jin-Mo Sung, Mustafa Zafer Karagozlu, JeaHyun Lee, Woori Kwak, Chang-Bae Kim

**Affiliations:** a Department of Life Science, Sangmyung University, Seoul, Korea;; b Bionics Co, Ltd, Seoul, Korea;; c C&K Genomics, Seoul, Korea

**Keywords:** Complete mitochondrial genome, gastropoda, *Menathais tuberosa*, Muricidae, phylogenetic tree

## Abstract

Complete mitochondrial genome of the knobbed rock shell sea snail *Menathais tuberosa* (Röding, 1798) has been sequenced and phylogenetic relationships evaluated due to mitochondrial protein coding genes. The size of mitochondrial genome for *M. tuberosa* is 15,294 bp and the nucleotide composition of the mitochondrial genome is 28.4% A, 16.5% C, 17.6% G and 37.5% T. Reconstructed phylogenetic tree of the Neogastropoda showed that *M. tuberosa* is in the monophyletic Muricidae. This is the first record of complete mitochondrial genome from the genus.

Muricidae is a large and diverse family of small to large predatory sea snails. There are 335 valid species in the family (WoRMS 2016). They are valuable ingredients of traditional medicines in China, India, South African and Middle Eastern countries (Benkendorff et al. 2015). There are only three complete mitochondrial genomes of the Muricidae species recorded in the Genbank: *Reishia clavigera* (Ki et al. 2010), *Rapana venosa* (Sun & Yang 2016) and *Bolinus brandaris* (Cunha et al. 2009). In this study, complete mitochondrial genome of *Menathais tuberosa* has been analyzed and reported for the first time. Furthermore, phylogenetic relationship of the species in the Neogastropoda was reconstructed based on amino acid sequences of mitochondrial protein coding genes.

The species were collected from the sandy bottom of Osakura Island, Chook Lagoon, Federated States of Micronesia (7°28′40″N, 151°53′49″E) on 07 July 2015 and preserved in 97% ethanol. The specimen was deposited in the Marine Biodiversity Institute of Korea (MABIK MO00157622). The size of mitochondrial genome for *M. tuberosa* (GenBank accession number KU747972) is 15,294 bp which is similar to the other species belonging to the same family, *R. clavigera* (15,285 bp), *R. venosa* (15,272 bp), *B. brandaris* (15,380 bp). AT content of the mitochondrial genome of the present species is 65.9%, which is lower than *R. clavigera* (66.2%), *R. venosa* (69.0%) and *B. brandaris* (66.4%). The mitochondrial genome is composed of 13 protein-coding genes, 2 ribosomal RNA genes, and 22 tRNA genes with 4 overlapping regions between 2 and 26 bp in length. Also, 27 intergenic sequences show length variation ranging from 1 to 56 bp and the largest intergenic sequence is located between NAD5 and tRNA-Phe genes. Gene orders also similar with complete mitogenome of the known Muricidae species, except *R. clavigera*. The phylogenetic study shows that *M. tuberosa* belongs to the Muricidae group and has sister relationship with the lineage including *R. clavigera* and *R. venosa* (Figure 1). Earlier reported mitochondrial genome based phylogenetic study of the Neogastropoda (Cunha et al. 2009) showed similar results. The present study provides additional data for Neogastropoda phylogeny.

**Figure 1.**
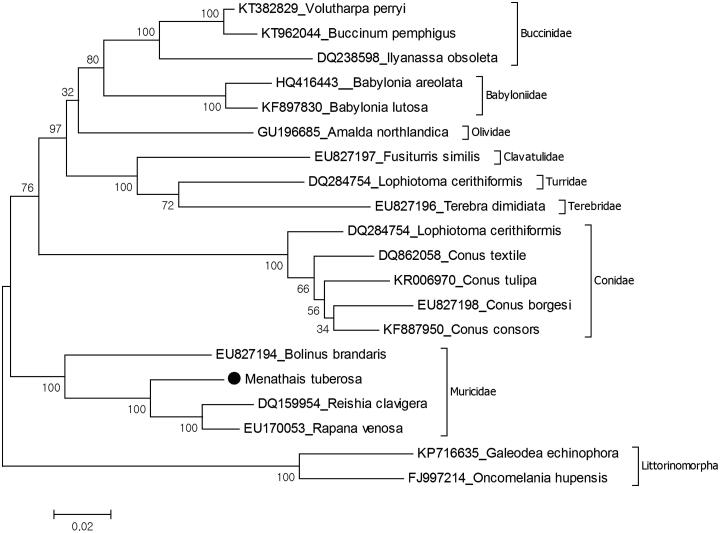
Molecular phylogeny of *Menathais tuberosa* in the Neogastropoda. The mitochondrial DNA was extracted and purified libraries were profiled using the Agilent Bioanalyzer and sequenced with the Illumina MiSeq platform to yield 300 bp paired end reads. Mitochondrial genes were assembled and annotated by MITObim software (Hahn et al. 2013) and MITOS web server (Bernt et al. 2013). The annotation of mitochondrial genome sequences was refined using Geneious software version 9.1.2 (http://www.geneious.com, Kearse et al. 2012). The phylogeny of *M. tuberosa* was reconstructed due to all protein-coding genes except ATP8 gene of mitochondrial genome with the maximum likelihood statistical method using MEGA 6.0 software (Tamura et al. 2013). mtREV with Freqs (+F) model used for amino acid substitution and bootstrap method replicated 1000 times for the test of phylogeny. The complete mitochondrial genomes of Neogastropoda and Littorinomorpha species were retrieved from the GenBank for reconstruction of the phylogenetic tree. Two species belonging to the order Littorinomorpha represent the outgroup.
